# Software Tools for Passive Acoustic Monitoring in Aquatic and Terrestrial Bio- and Ecoacoustics: A Living Systematic Review

**DOI:** 10.12688/f1000research.173495.1

**Published:** 2026-01-13

**Authors:** Tara Hanf-Dressler, Rym Nouioua, Karolin Thomisch, Dorian Cazau, Simon de Selys Longchamps, Kevin F.A. Darras

**Affiliations:** 1 Evolutionary Ecology, Leibniz Institute for Zoo and Wildlife Research, Berlin, 10315, Germany; 2 Botany and Biodiversity Research, Faculty of Life Sciences, University of Vienna, Vienna, 1030, Austria; 3 Ocean Acoustics Group, Alfred Wegener Institute, Helmholtz Centre for Polar and Marine Research, Bremerhaven, 27515, Germany; 4 CNRS Lab-STICC, ENSTA, Institut Polytechnique de Paris, Brest, 29200, France; 5 INRAE, EFNO, Nogent-sur-Vernisson, 45290, France

**Keywords:** acoustic data processing, audio analysis, bioacoustics, ecology, PAM workflow, soundscape

## Abstract

Biodiversity monitoring is crucial for understanding species trends and their responses to anthropogenic change. Passive acoustic monitoring (PAM) offers a scalable, non-invasive approach to capture ecological information across large spatial and temporal scales. However, it generates vast amounts of audio recordings, whose management and analysis present technical challenges. To support diverse user needs in ecoacoustic research, a growing number of software tools have emerged, but the landscape remains fragmented and difficult to navigate. We provide a systematic overview of software tools used for soundscape assessment across terrestrial, freshwater, and marine environments. We screened peer-reviewed literature and complemented it with database cross-checking to identify and categorize tools according to four PAM data workflow components: data management, signal pre-processing, visualisation and navigation, and acoustic analysis. We found 221 available tools of which 174 were explicitly designed for PAM. Most tools were freely accessible (83%) with only a smaller fraction being commercial (12%) or limited access (5%). Terrestrial research accounted for most software mentions (476 studies), followed by aquatic (319) and cross-realm (64) studies. Nearly half (45%) were package-based frameworks within R, Python, or MATLAB. Acoustic analysis was the most represented workflow component, while only 40 tools covered all four of them. This diversity illustrates the field’s rapid technical growth but also its redundancy and methodological fragmentation: to date, many tools target only a subset of workflow components and replicate similar functionalities. Despite this, the prevalence of PAM-dedicated software indicates increasing specialization and technical maturity within ecoacoustics. Our structured inventory underscores the need for greater collaboration and continuity in software development, promoting the improvement and accessibility of existing tools rather than further proliferation. This living systematic review, provides a practical, biannually updated reference for tool selection and fosters transparency, comparability, and cooperation across bioacoustic and ecoacoustic research communities.

## 1. Introduction

Monitoring biodiversity under accelerating global change requires scalable and non-invasive technologies capable of recording ecological dynamics continuously and comparably across broad spatial and temporal scales. One such method is passive acoustic monitoring (PAM), which has emerged as a transformative approach for collecting ecological data ( Au and Hastings, 2008; Ross et al., 2023), enabling researchers to survey ecosystems by recording, storing, pre-processing, and analysing environmental sounds ( Pijanowski et al., 2024; Sugai et al., 2019; Gibb et al. 2019; Mooney et al., 2020; Bolgan et al., 2023). PAM offers key advantages for biodiversity assessment: it allows for continuous, long-term, and large-scale monitoring of acoustic activity patterns, community-level dynamics, soundscapes, and environmental change while minimizing disturbance to natural systems ( Farina, 2014; Ross et al., 2023). It facilitates standardized, repeatable observations even in remote or inaccessible regions across aquatic or terrestrial realms (e.g., Burivalova et al. 2019; Elise et al., 2019; Sethi et al., 2020).

Within this methodological framework, the terms bioacoustics and ecoacoustics are often used variably across disciplines and geographic contexts, sometimes even synonymously with PAM ( Au and Hastings, 2008; Sueur & Farina, 2015; Penar et al., 2020; Pijanowski et al., 2024). Both terms frame PAM as a unifying methodological backbone that links acoustic data to biodiversity patterns ( Sueur et al., 2008), assessing impacts of disturbances on ecosystems and wildlife ( Gitau et al., 2025) as well as climate change ( Krause & Farina, 2016; Sueur et al., 2019), and informing land management decisions ( Ross et al., 2023; Cord et al., 2025).

However, implementation of PAM in ecological research is constrained by several limitations. Automated recorders produce vast amounts of data per study, often referred to as “big data” ( Sagiroglu and Sinanc, 2013; Kowarski and Moors-Murphy, 2021), posing substantial challenges for storage, management, pre-processing and analysis of acoustic data ( Sugai et al., 2019; Pijanowski et al., 2024). A wide range of software tools has been developed to support PAM data workflows, yet they are not always freely accessible or standardized across research domains, taxa, or geographic realms. This diversity often mirrors methodological divergence rather than coordinated progress ( Darras et al., 2023; Roch et al., 2016; Wieczorek et al., 2012). Software tool fragmentation occurs both at the functional level, where tools differ in design, purpose, and compatibility, and at the community level, where limited awareness and lack of centralized guidance lead researchers to rely on familiar or locally established software.

Several recent reviews have outlined methodological advances and conceptual frameworks in response to the increasing demand for methodological guidance: they proposed structured PAM workflows spanning data acquisition, signal pre-processing, detection, classification, and interpretation, and discussed how different tools fit within these functionality classes (e.g., Ducrettet et al., 2025; Kvsn et al., 2020; Lindseth and Lobel, 2018; Ross et al., 2023; Rhinehart et al., 2020; Sugai et al., 2020; Wall et al., 2025). Others focused on algorithmic approaches and classification methods, including traditional template matching and decision-tree approaches as well as machine-learning and deep-learning techniques ( Knight et al., 2017; Priyadarshani et al., 2018; Stowell, 2022; Usman et al., 2020). Together, these studies provide valuable overviews of PAM workflows and analytical strategies. However, they remain largely conceptual and method-focused, and do not offer a systematic, data-centred inventory of available software tools across PAM workflows. Yet, the sheer variety of analytical approaches can be bewildering, reflecting both the creative adaptability of the field and its ongoing search for conceptual coherence. Lists of software tools managing acoustic data such as the Bioacoustics Software Database ( Rhinehart et al., 2024), the acoustic monitoring guide provided by WWF UK ( Browning et al., 2017) or entries on Wikipedia ( “List of bioacoustics software”, 2024), provide an important starting point, yet none offers a systematic complete and comprehensive synthesis of software tools and their functional coverage across the entire ecoacoustic workflows.

To address this gap, we undertook a two-step approach. First, we conducted a systematic screening of peer-reviewed literature in the fields of bio- and ecoacoustics across aquatic and terrestrial realms to identify studies that explicitly use software tools for PAM. Second, based on this literature review, we compiled a comprehensive list of software tools, informed by additional sources such as the Bioacoustics Software Database ( Rhinehart et al., 2024) and expert knowledge. Building on previously proposed workflow classifications (e.g., Ross et al., 2023; Rhinehart et al., 2020; Sugai et al., 2020), we refined the structure to capture the full range of functionalities implemented in ecoacoustic software, reflecting the typical progression from raw data handling to interpretation in ecoacoustic studies. We defined four workflow components of ecoacoustic data processing that do not represent a strict sequential order but rather categories of functionalities that are commonly used in PAM: (1) data management, (2) signal pre-processing, (3) visualisation and navigation, and (4) acoustic analysis. This functional classification is intended to help to navigate the software landscape and identify tools suited to user needs across both aquatic and terrestrial realms. This literature-based review, which will be updated on a biannual basis, aims to advance transparency, reproducibility, and collaboration in ecoacoustic and bioacoustic research and to serve as a lasting, practical reference for the scientific community.

## 2. Methods

### 2.1 Literature search

We performed a systematic review of peer-reviewed literature, following the Preferred Reporting Items for Systematic Reviews and Meta-Analyses (PRISMA) 2020 guidelines ( Page et al., 2021, Figure 1). We used the Web of Science (WoS) Core Collection for our literature search, as it provides consistent indexing across relevant disciplines. We limited the search to studies published between 2014-01-01 and 2024-01-01 to exclude discontinued software tools. The search strategy was developed iteratively by testing and refining keyword combinations to improve the specificity and relevance of the results. We included the terms *bioacoustics* and *ecoacoustics* in our search strategy because both are commonly associated with PAM and often overlap in scope and terminology ( Sueur & Farina, 2015; Penar et al., 2020; Pijanowski et al., 2024). Since studies from each field frequently reference PAM software and analytical workflows, including both terms ensured a comprehensive representation of relevant literature across ecological realms and research domains. The final search string was as follows:

**Figure 1.**
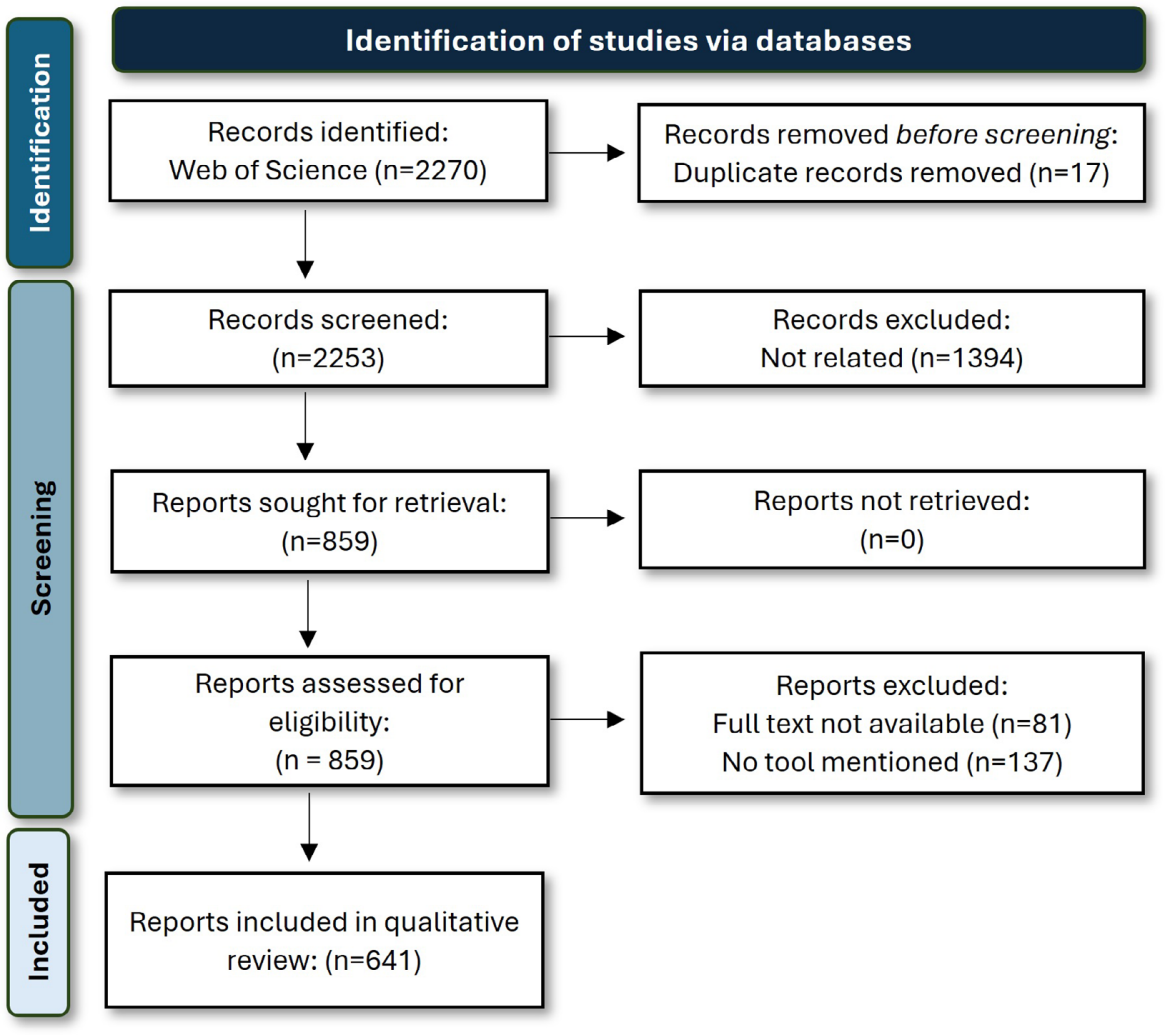
PRISMA flow diagram summarizing the literature screening and selection process ( Page et al., 2021). The diagram illustrates the number of records identified, screened, and included in the review, as well as reasons for exclusion at each stage. A total of 2270 records were initially identified through database searches, of which 1394 were excluded after title and abstract screening. After full-text assessment, 641 studies were retained for inclusion. Only studies explicitly reporting the use of software tools in bioacoustic or ecoacoustic research were included.

TS = ((software OR application OR platform OR program OR package) AND (ecoacoustic* OR “eco-acoustic*” OR bioacoustic* OR “bio-acoustic*” OR soundscape* OR “passive acoustic monitor*” OR “passive acoustic record*” OR “passive acoustic sens*” OR “auto* sound record*” OR “auto* audio record*” OR “auto* acoustic record*” OR “auto* record* unit”))

The final query returned 2,270 records from the Web of Science database. After removing 17 duplicate entries, 2,253 records were screened by title and abstract by all co-authors to assess their relevance to ecoacoustic and bioacoustic research. Studies employing PAM in disciplines outside the scope of bio- or ecoacoustics were excluded, even if similar recording techniques were used. Specifically, we excluded studies in the field of medicine (e.g., biomedical acoustic signal analysis), physics (e.g., acoustic wave propagation), indoor acoustics, urban soundscape design, and human sound perception, as these have non-ecological contexts and apply different methodological frameworks. Following this screening, 859 studies were retained for full-text assessment ( Figure 1). We included all studies within the fields of bioacoustics and ecoacoustics, defined broadly to encompass applications of PAM for the investigation of ecological patterns, biodiversity, species presence, and soundscape dynamics across multiple taxa as well as aquatic (marine and freshwater) and terrestrial realms. We identified the reported use of software tools in relevant studies and excluded those that did not mention any. Because the search did not include full-text keyword screening, our inventory likely underrepresents the total number of software tools, as those mentioned exclusively within the main text would not have been captured. This limitation reflects a common challenge in literature-based software reviews and should be considered when interpreting coverage completeness. We further classified the relevant studies into aquatic or terrestrial realms to identify their contexts of application.

### 2.2 Data extraction and functional characterization of software tools

We define *software* as a computer program or application designed for direct interaction by end users, excluding operating systems and system-level utilities ( Fair et al., 2005). In this review, this definition includes any digital application supporting one or more components of the PAM workflow (data management; signal pre-processing; visualisation and navigation; acoustic analysis), encompassing stand-alone tools, online platforms, and packages integrated into programming environments such as R, MATLAB, or Python. Publicly accessible AI models (e.g., trained neural networks, automated classifiers) and researcher-developed scripts were also included and treated as software tools when they implemented specific analytical functions such as detection, classification, or feature extraction within PAM workflows. We deliberately adopt a broad definition to capture both widely distributed tools and accessible researcher-developed solutions, as both play a central role in the current ecoacoustic research landscape. Unavailable or undocumented custom scripts that were project-specific or written for internal use were grouped into a single category and excluded from subsequent analyses of software functionalities. These scripts were written in various programming environments (e.g., R, MATLAB, Python) but lacked standardized documentation.

For each available software tool, we systematically extracted information from peer-reviewed publications, official software documentation, user manuals, and publicly available developer resources. We extracted general software metadata including: software availability (yes/no), access type (free, free to try, or commercial), deployment form (local installation, web-based, or package within another software environment), execution (the higher-level software or programming environment in which the tool operates), PAM-dedication (tool developed exclusively for PAM or not), geographical realm (aquatic, terrestrial, or general), and taxa (targeted taxonomic groups, or general) ( Table 1).

**Table 1.** Description of extracted functional and metadata information of software tools.

	NAME	DEFINITION
**SOFTWARE METADATA**	**Availability**	Whether a software can be obtained online, via download or purchase in 2025.
	**Access**	Defines whether the software is fully free, partially free (e.g., free to try or freemium), or requires payment (commercial).
	**Deployment**	Whether software is running on local machines (local), on web-servers (online) or integrated within other, usually local software (package).
	**Execution**	Specifies the environment in which the software runs, such as an operating system (OS) or within a hosting software (e.g., R, MATLAB or Python).
	**PAM-dedicated **	Whether a software tool was exclusively developed for passive acoustic monitoring (PAM) workflow in environmental or ecological research.
	**Realm**	Primary environmental context for which the software tool is intended, such as aquatic, terrestrial, or general.
	**Taxa**	Broad taxonomic group(s) targeted by the software, based on IUCN Red List taxonomy.
**PAM WORKFLOW COMPONENTS**	**Data management (DM)**	Functionalities to manage recordings or their metadata.
	**Signal pre-processing (SP)**	Digital manipulation of audio signals, including modification and synthesis.
	**Visualization & navigation (VN)**	Functionalities for representing audio signals graphically and finding target signals by listening.
	**Acoustic analysis (AA)**	Functionalities for extracting meaning from/interpreting audio signals.

**Figure 2.**
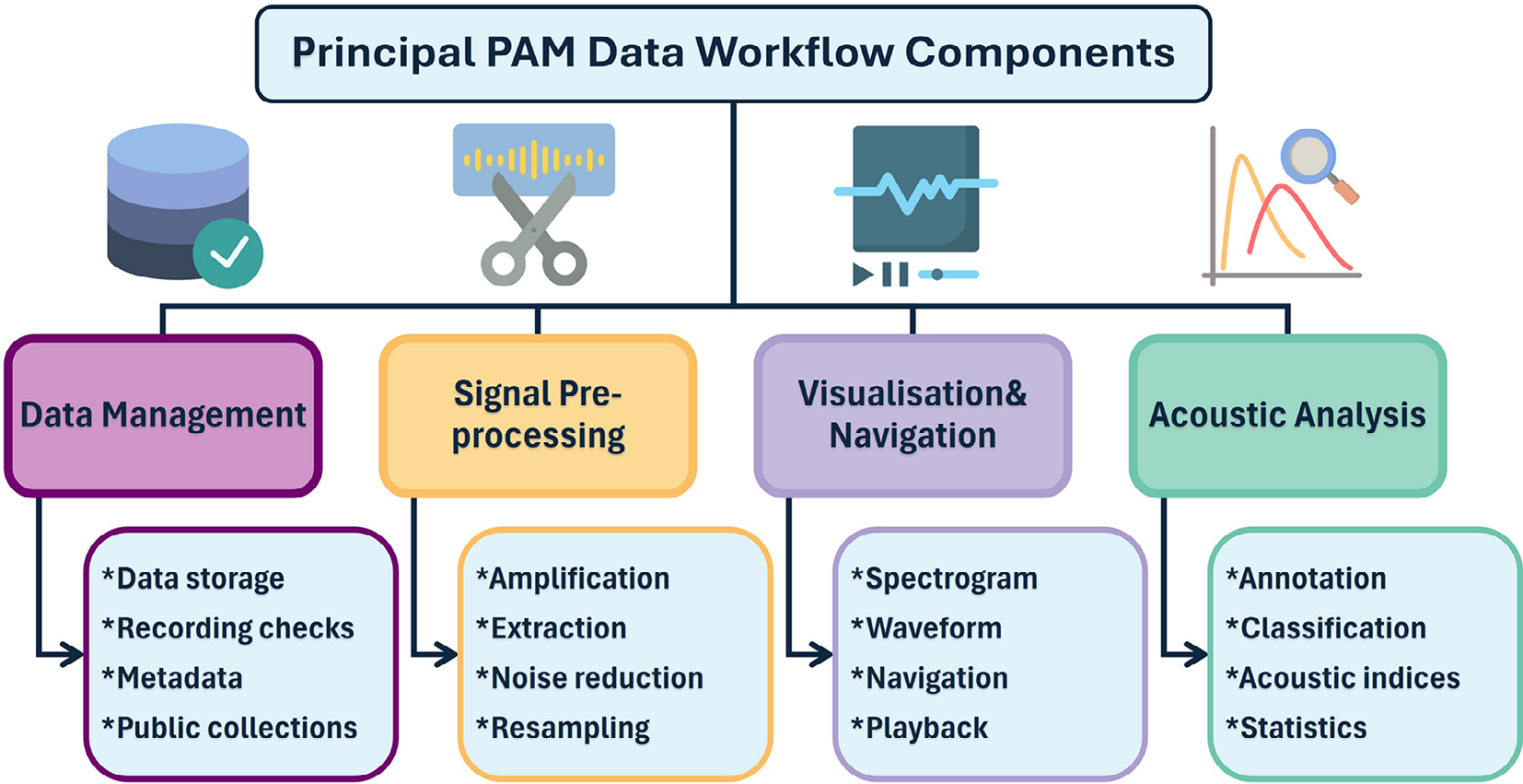
Overview of four common PAM data workflow components in acoustic data processing, with examples for each. These categories form the basis for assessing the functional coverage of different software tools in ecoacoustic research. The listed functionalities are non-exhaustive and serve as examples. Icons by Flaticon, 2025.

Based on previous reviews, we defined four principal components of PAM data workflows (e.g., Pijanowski et al., 2024; Priyadarshani et al., 2018; Stowell 2022) (see Figure 2): (1) data management (including functionalities such as data storage, recording checks, spatial and temporal metadata, public collections, etc.), (2) signal pre-processing (e.g., amplification, segment extraction, noise reduction or resampling of acoustic data), (3) spectrogram visualisation and navigation (e.g., spectrogram, waveform, audio navigation, playback), (4) acoustic analysis (e.g., annotation, classification, calculation of acoustic indices or statistics). These are not necessarily sequential workflow stages in a PAM data processing as they can be applied independently or in combination. In this initial version of the living systematic review, we provide a broad overview of principal workflow components covered by each software tool. Their definitions are mainly determined by the (sub-)functionalities that they contain; future versions of this review will expand on this by detailing specific functions within each functionality component. To maintain the living and community-driven nature of this review, we encourage researchers to contact the authors with new software tools, projects, or updates that could be incorporated in subsequent versions.

In addition to the literature screening, we incorporated software tools identified through an internal review by the co-author team, which includes terrestrial and marine ecologists as well as specialists of soniferous taxa (birds, bats, and mammals). To ensure broad coverage, the resulting tool list was cross-checked against publicly available resources, including the Bioacoustics Software Database ( Rhinehart et al., 2024) and Wikipedia ( “List of bioacoustics software”, 2024).

### 2.3 Data analysis

We performed all statistical analyses in *R v4.4.0* ( R Core Team, 2024). We first harmonised naming conventions by merging duplicate entries, consolidating alternative spellings and excluded unavailable tools (e.g., no longer accessible, or lacking documentation at the time of analysis). The number of software tools was analysed according to available metadata and workflow components. Visualizations were produced using ggplot2 ( Wickham, 2016) for graphing and other *tidyverse* packages ( Wickham et al., 2019) for data handling and preparation. To assess the overlap of core functionalities across software tools, we conducted a cross-feature analysis using the *UpSetR* package ( Conway et al., 2017). This approach allowed us to visualize the intersections between tools offering functionalities in data management, signal pre-processing, visualization & navigation, and acoustic analysis. In addition, we created an interactive dendrogram using the *collapsibleTree* package ( Khan et al., 2023), enabling users to identify and compare software tools according to their functional attributes and analytical scope. The complete script and dendrogram can be accessed here: https://doi.org/10.17605/OSF.IO/V72CE ( Hanf-Dressler et al., 2025).

## 3. Results and discussion

### 3.1 Diversity and fragmentation across software tools

**Figure 3.**
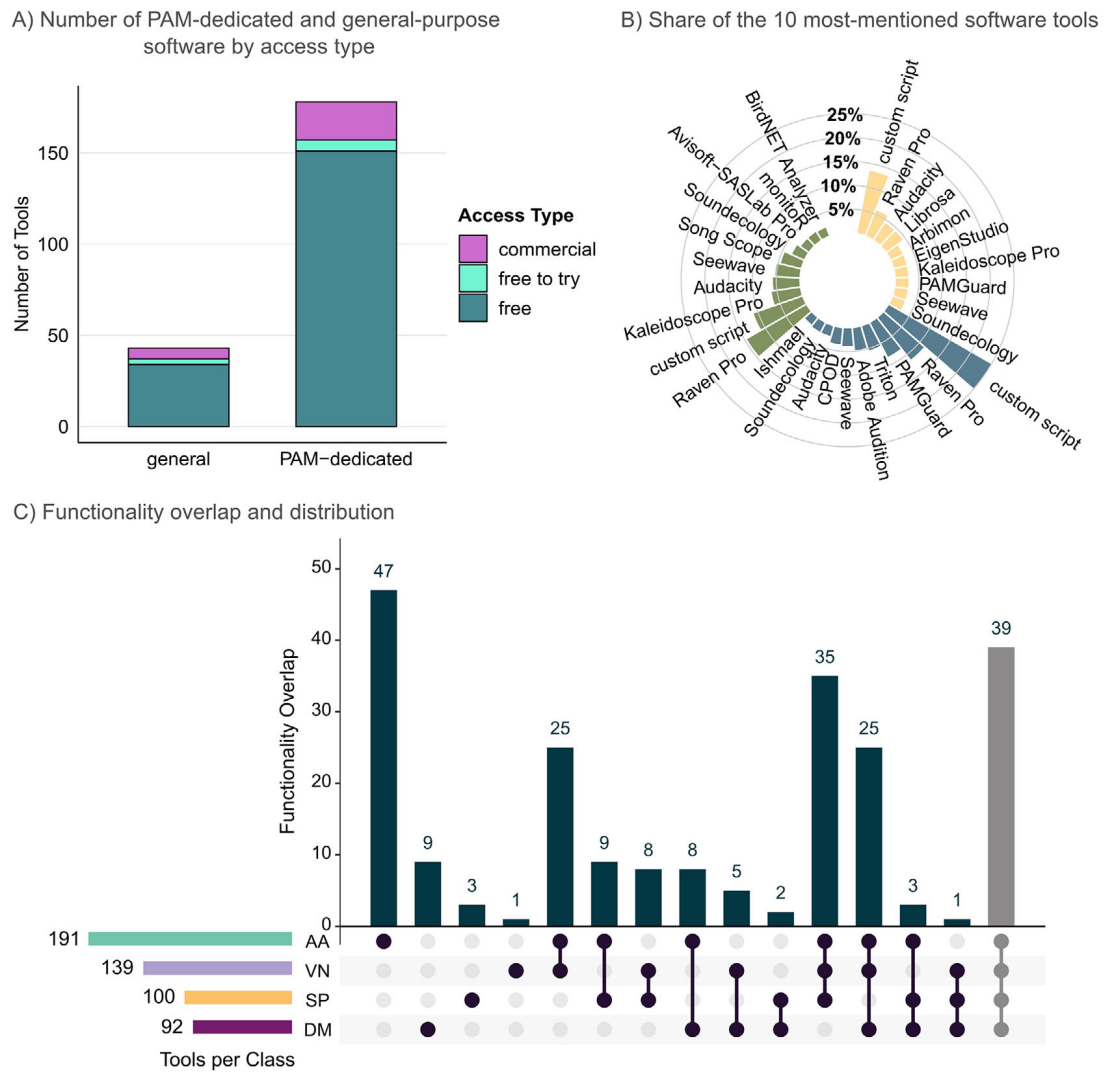
Reviewed software tools across the PAM workflow: (A) Number of PAM-dedicated and general-purpose software, categorized by access type (commercial, free to try, free). (B) Percentages of top 10 most frequently mentioned software within terrestrial (n = 647), aquatic (n = 321) or general (cross-realm) studies (n = 66). Percentages are based on the total number of explicit software mentions in peer-reviewed publications for each ecological realm. (C) Functionality overlap among software tools: DM = Data management, SP = Signal pre-processing, VN = Visualisation & navigation, AA = Acoustic analysis (colour coding follows Figure 2). Tools implementing all four workflow components are highlighted in grey. Custom scripts were excluded from panels B–C.

Our systematic literature screening identified 189 distinct software tools used in ecoacoustic and bioacoustic studies. We further complemented the list with 66 additional tools (identified through expert input and database cross-checking), including 38 retrieved from the Bioacoustics Software Database ( Rhinehart et al., 2024). This resulted in a list of 251 tools, of which 221 were available at the time of analysis (excluding custom scripts). Among these, 178 tools were specifically designed for PAM ( Figure 3A), illustrating both the rapid technological expansion and methodological maturity of ecoacoustic research. Most software was open-source (free; PAM-dedicated software = 151; non-dedicated software = 34), with only a few commercial (PAM-dedicated = 21; non-dedicated = 6) or free-to-try tools (PAM-dedicated = 6; non-dedicated = 3), indicating a strong open-access orientation within the community. The full inventory, including key metadata and functional workflow components, is provided in Table 2, alongside an interactive dendrogram visualising the software landscape by functionality and general characteristics available here: https://doi.org/10.17605/OSF.IO/V72CE ( Hanf-Dressler et al., 2025).

**Table 2.** Overview of available software tools used for passive acoustic monitoring and their coverage of four common functional workflow components: DM = data management, SP = Signal pre-processing, VN = visualisation and navigation, AA = Acoustic Analysis.

NAME	PAM-DEDICATED	ACCESS	DEPLOYMENT	EXECUTION	TAXA	DM	SP	VN	AA
*A2O*	YES	free	online	web browser	Generalist/multiple taxa	YES	NA	NA	YES
*Acoular*	NO	free	package	Python	Generalist/multiple taxa	NA	YES	YES	YES
*Acoustic Toolbox User Interface and Post Processor*	NO	free	package	Matlab	Generalist/multiple taxa	NA	NA	NA	YES
*Acoustic Workbench*	YES	free	online	web browser	Generalist/multiple taxa	YES	YES	YES	YES
*Adobe Audition*	NO	commercial	local	Windows, MacOS	Generalist/multiple taxa	NA	YES	YES	YES
*AlexNet*	NO	free	package	Matlab	Generalist/multiple taxa	NA	NA	NA	YES
*AMMonitor*	YES	free	local	R	Generalist/multiple taxa	YES	NA	NA	YES
*Anabat Insight*	YES	commercial	local	Windows, MacOS	Chiroptera	YES	YES	YES	YES
*Animal Sound Identifier*	YES	free	package	Matlab	Generalist/multiple taxa	NA	NA	YES	YES
*AnimalSpot*	YES	free	local	Windows,MacOS, Linux	Generalist/multiple taxa	NA	YES	YES	YES
*APLOSE*	YES	free	online	web browser	Certartiodactyla/Pinnipedia	YES	NA	YES	YES
*Arbimon*	YES	free	online	web browser	Generalist/multiple taxa	YES	YES	YES	YES
*ARBIMON touch*	YES	free	local	Android	Generalist/multiple taxa	YES	NA	YES	YES
*ArcticBirdSounds*	YES	free to try	online	web browser	Aves	YES	NA	NA	NA
*ArtemiS SUITE*	NO	commercial	local	Windows	Generalist/multiple taxa	NA	YES	YES	YES
*ARTwarp*	YES	free	package	Matlab	Generalist/multiple taxa	NA	NA	NA	YES
*Audacity*	NO	free	local	Windows, MacOS, Linux	Generalist/multiple taxa	NA	YES	YES	YES
*AudioDB*	YES	free	local	Ubuntu, Windows	Generalist/multiple taxa	YES	NA	YES	YES
*AudioMoth-Scripts *	YES	free	local	Python	Generalist/multiple taxa	YES	NA	NA	NA
*audioset-soundscape-feat*	YES	free	package	Python	Generalist/multiple taxa	NA	NA	YES	YES
*AVA*	YES	free	package	Python	Generalist/multiple taxa	NA	YES	YES	YES
*AVES*	YES	free	package	Python	Aves	NA	NA	NA	YES
*AVGN*	YES	free	package	Python	Aves	NA	YES	NA	YES
*AviaNZ*	YES	free	local	Windows,MacOS, Linux	Aves	NA	YES	YES	YES
*Avisoft Recorder USG software*	YES	free to try	local	Windows, Linux, MacOS	Generalist/multiple taxa	YES	YES	YES	YES
*Avisoft-SASLab Lite*	YES	commercial	local	Windows	Generalist/multiple taxa	YES	YES	YES	YES
*Avisoft-SASLab Pro*	YES	commercial	local	Windows	Generalist/multiple taxa	YES	YES	YES	YES
*bacpipe*	YES	free	package	Python	Generalist/multiple taxa	NA	NA	NA	YES
*Banter*	YES	free	package	R	Certartiodactyla/Pinnipedia	NA	NA	NA	YES
*BAT*	YES	free	local	Python	Chiroptera	NA	NA	YES	YES
*Batclassify*	YES	free	package	Python	Chiroptera	NA	YES	YES	YES
*BatDetect2*	YES	free	package	Python	Chiroptera	NA	NA	NA	YES
*BatExplorer*	YES	commercial	local	Windows	Chiroptera	YES	YES	YES	YES
*batIdent*	YES	free	local	MacOS	Chiroptera	NA	NA	NA	YES
*BatNet*	YES	free	package	Python	Chiroptera	NA	NA	NA	YES
*BatRack*	YES	free	package	Python	Chiroptera	NA	NA	NA	YES
*BatScope*	YES	commercial	local	Windows, macOS	Chiroptera	YES	YES	YES	YES
*BATscreen Pro*	YES	commercial	local	Windows	Chiroptera	YES	YES	YES	YES
*BatSound Pro*	YES	commercial	local	Windows	Chiroptera	YES	YES	YES	YES
*bcAdmin*	YES	commercial	local	MacOS	Chiroptera	YES	YES	YES	YES
*bcAnalyze*	YES	commercial	local	MacOS	Chiroptera	YES	NA	YES	YES
*BCID Canada*	YES	commercial	local	Windows	Aves	YES	YES	NA	YES
*BCID Eastern USA*	YES	commercial	local	Windows	Aves	YES	YES	NA	YES
*BCID United Kingdom*	YES	commercial	local	Windows	Aves	YES	YES	NA	YES
*Bellhop*	NO	free	package	Fortran, Matlab, Python	Generalist/multiple taxa	NA	NA	NA	YES
*Beluga*	YES	free	local	Matlab, Windows	Certartiodactyla/Pinnipedia	NA	YES	YES	YES
*BioAcoustic Index Tool (BAIT)*	YES	free	package	R	Generalist/multiple taxa	NA	YES	NA	YES
*Bioacoustic Workbench*	YES	free	online	web browser	Generalist/multiple taxa	YES	YES	YES	YES
*BioAcoustica*	YES	free	online	Web browser	Generalist/multiple taxa	YES	YES	YES	NA
*bioacoustics*	YES	free	package	R	Generalist/multiple taxa	NA	YES	YES	YES
*Bioacoustics Model Zoo*	YES	free	package	Python	Generalist/multiple taxa	NA	NA	NA	YES
*BioCPPNet*	YES	free	package	Python	Generalist/multiple taxa	NA	YES	NA	YES
*BioLingual*	YES	free	package	Python	Generalist/multiple taxa	NA	NA	NA	YES
*Bird Sounds Global web*	YES	free	online	web browser	Aves	YES	NA	YES	YES
*BirdNET Analyzer*	YES	free	online	Windows, MacOS	Aves	YES	NA	YES	YES
*BirdNET SoundID App*	YES	free	online	Android App, iOS app	Aves	YES	NA	YES	YES
*BirdNET-Annotator *	YES	free	package	Docker	Aves	NA	NA	NA	YES
*BirdVoxClassify*	YES	free	package	Python	Aves	NA	NA	NA	YES
*BirdVoxDetect*	YES	free	package	Python	Aves	NA	NA	NA	YES
*BTO acoustic pipeline*	YES	free to try	online	web browser	Generalist/multiple taxa	YES	YES	YES	YES
*CadnaA*	NO	commercial	local	Windows	Generalist/multiple taxa	YES	NA	NA	YES
*callViewer*	YES	free	package	Matlab	Chiroptera	NA	NA	NA	YES
*caret*	NO	free	package	R	Generalist/multiple taxa	NA	NA	NA	YES
*CASE*	YES	free	package	Matlab	Generalist/multiple taxa	NA	NA	YES	YES
*ChiroVox*	YES	free	online	web browser	Chiroptera	YES	NA	NA	NA
*Chirpity*	YES	free	local	Windows, MacOS, Linux	Insecta	YES	YES	YES	YES
*CHORUS*	YES	free	local	Matlab/Windows	Generalist/multiple taxa	YES	YES	YES	YES
*CityNET*	YES	free	package	Python	Generalist/multiple taxa	NA	YES	NA	YES
*Cool Edit Pro*	NO	free to try	local	Windows	Generalist/multiple taxa	NA	YES	YES	NA
*CPOD*	YES	free	local	Windows	Certartiodactyla/Pinnipedia	YES	YES	YES	YES
*crowsetta*	YES	free	package	Python	Generalist/multiple taxa	NA	NA	NA	YES
*DAS*	YES	free	package	Python	Generalist/multiple taxa	NA	YES	YES	YES
*DAS4Whales*	YES	free	package	Python	Certartiodactyla/Pinnipedia	YES	YES	YES	YES
*Dawn Chorus*	YES	free	online	Android, iOS	Aves	YES	NA	NA	YES
*dBWav software*	YES	commercial	local	Windows	Generalist/multiple taxa	YES	NA	YES	YES
*deep whistle contour (DWC) detector*	YES	free	package	Python, Matlab	Certartiodactyla/Pinnipedia	NA	NA	YES	YES
*DeepSqueak*	YES	free	package	Matlab	Certartiodactyla/Pinnipedia	NA	YES	YES	YES
*DetEdit*	YES	free	package	Matlab	Generalist/multiple taxa	NA	NA	YES	YES
*eBird*	YES	free	online	web browser, Android, iOS	Aves	YES	NA	NA	YES
*Echo Meter Touch companion App*	YES	commercial	local	Android, iOS	Chiroptera	YES	NA	YES	YES
*Echodash*	YES	free to try	online	web browser	Aves	YES	NA	YES	NA
*Echoview*	NO	commercial	local	Windows	Generalist/multiple taxa	NA	YES	YES	YES
*EcoAcoustic Bank*	YES	free	online	web browser	Generalist/multiple taxa	YES	NA	NA	YES
*Ecoacoustics Audio Analysis Software*	YES	free	local	Windows, Unix, MacOS	Generalist/multiple taxa	NA	NA	NA	YES
*Ecophony*	YES	free	online	web browser	Generalist/multiple taxa	YES	NA	NA	NA
*ecoSound-web *	YES	free	online	web browser, Docker	Generalist/multiple taxa	YES	YES	YES	YES
*EigenStudio*	NO	free	local	Windows	Generalist/multiple taxa	YES	NA	NA	NA
*Essentia*	NO	free	local	Python	Generalist/multiple taxa	NA	YES	YES	YES
*F-POD software*	YES	free	local	Windows	Certartiodactyla/Pinnipedia	YES	YES	YES	YES
*FishSounds*	YES	free	online	web browser	Actinopterygii/other fishes	YES	NA	YES	NA
*FrogID*	YES	free	online	Android, iOS	Amphibia	YES	NA	NA	YES
*Gemini SeaTec*	NO	commercial	local	NA	Certartiodactyla/Pinnipedia	NA	NA	NA	YES
*gibbonR*	YES	free	package	R	Generalist/multiple taxa	NA	NA	NA	YES
*GlassOFire*	YES	free	local	Windows	Aves	NA	YES	YES	NA
*GoldWave*	NO	free to try	local	Windows, Linux, MacOS	Generalist/multiple taxa	NA	YES	YES	NA
*hardRain*	YES	free	package	R	Generalist/multiple taxa	NA	NA	NA	YES
*HARK/HARKBird*	YES	free	package	Python	Aves	YES	NA	YES	YES
*HawkEars*	YES	free	package	Python	Aves	NA	NA	NA	YES
*Hidden Markov Model toolkit*	NO	free	local	Linux, Windows	Generalist/multiple taxa	NA	NA	NA	YES
*hybrid-vocal-classifier*	YES	free	package	Python	Generalist/multiple taxa	NA	NA	NA	YES
*iBatsID*	YES	free	online	web browser	Chiroptera	NA	NA	YES	YES
*Ishmael*	YES	free	local	Windows	Certartiodactyla/Pinnipedia	NA	YES	YES	YES
*Kaldi*	NO	free	local	Windows	Generalist/multiple taxa	NA	NA	NA	YES
*Kaleidoscope Lite*	YES	free	local	Windows, Linux, MacOS	Generalist/multiple taxa	YES	YES	YES	YES
*Kaleidoscope Pro*	YES	commercial	local	Windows, Linux, MacOS	Generalist/multiple taxa	YES	YES	YES	YES
*Ketos*	YES	free	package	Python	Generalist/multiple taxa	YES	YES	YES	YES
*KOE*	YES	free	online	web browser	Aves	YES	YES	YES	YES
*LAME*	NO	free	local	Windows, GNU/Linux, MacOS, Unix, other	Generalist/multiple taxa	NA	YES	NA	NA
*LASER (Localize Animal Sound Events Reliably)*	YES	free	package	Matlab	Generalist/multiple taxa	YES	YES	YES	YES
*LEAVES*	YES	free	package	Python, Windows, MacOS	Generalist/multiple taxa	NA	YES	YES	YES
*LF detection and classification system (LFDCS)*	YES	free	package	IDL on MacOS	Certartiodactyla/Pinnipedia	NA	NA	YES	YES
*Librosa*	NO	free	package	Python	Generalist/multiple taxa	NA	YES	YES	NA
*Low Frequency Detection and Classification System (LFDCS)*	YES	free	local	MacOS, IDL	Certartiodactyla/Pinnipedia	NA	YES	NA	YES
*Luscinia*	YES	free	local	Windows, Linux, MacOS	Generalist/multiple taxa	YES	NA	YES	YES
*Macaulay Library (Cornell Lab of Ornithology)*	YES	free	online	web browser	Generalist/multiple taxa	YES	NA	YES	YES
*MANTA*	YES	free	local	Windows, MacOS	Generalist/multiple taxa	YES	NO	YES	YES
*MATLAB (custom code)*	NO			Matlab		NA	NA	NA	NA
*Matlab SonoScape*	YES	free	package	Matlab	Generalist/multiple taxa	NA	NA	NA	YES
*Merlin Bird ID*	YES	free	local	Android, I	Aves	YES	YES	YES	YES
*Mice-USVs-segmentation-and-classification*	YES	free	package	Matlab	Other Mammalia	NA	YES	YES	YES
*MIRToolbox*	NO	free	package	Matlab	Generalist/multiple taxa	NA	NA	NA	YES
*monitoR*	YES	free	package	R	Generalist/multiple taxa	YES	NA	YES	YES
*monitoraSOM*	YES	free	package	R	Generalist/multiple taxa	YES	YES	YES	YES
*Mupet*	YES	free	package	Matlab	Other Mammalia	NA	YES	YES	YES
*NABat ML*	YES	free	online	web browser	Chiroptera	NA	NA	YES	YES
*NARW detection tool*	YES	free	package	Python	Certartiodactyla/Pinnipedia	NA	NA	YES	YES
*NEAL+*	YES	free to try	package	R	Aves	YES	NA	YES	YES
*NightHawk*	YES	free	package	Python	Aves	NA	NA	NA	YES
*Ocenaudio*	NO	free	local	Windows, MacOS, Linux	Generalist/multiple taxa	NA	YES	YES	NA
*Ohun*	YES	free	package	R	Generalist/multiple taxa	NA	NA	YES	YES
*OpenEcho*	YES	free	local	Python	Chiroptera	YES	NA	YES	YES
*OpenSoundscape*	YES	free	package	Python	Generalist/multiple taxa	NA	YES	YES	YES
*OPUS*	YES	free	online	web browser	Generalist/multiple taxa	YES	YES	YES	YES
*ORCA-SPOT *	YES	free	package	Python	Certartiodactyla/Pinnipedia	NA	NA	NA	YES
*ORCA-SPY *	YES	free	package	PAMGuard	Certartiodactyla/Pinnipedia	NA	YES	NA	YES
*Ornitho*	YES	free to try	online	web browser	Aves	YES	NA	YES	YES
*Osprey*	NO	free	package	Matlab	Generalist/multiple taxa	NA	NA	YES	NA
*other (e.g. custom CNNs)*	NO			NA		NA	NA	NA	NA
*PAMGuard*	YES	free	local	Windows, Linux, MacOS	Generalist/multiple taxa	YES	YES	YES	YES
*PAMGuide*	YES	free	package	R, Matlab	Certartiodactyla/Pinnipedia	NA	NA	YES	YES
*PAMlab*	YES	commercial	local	Windows, MacOS, Linux	Certartiodactyla/Pinnipedia	YES	YES	YES	YES
*PAMpal*	YES	free	package	R	Generalist/multiple taxa	YES	NO	YES	YES
*paPAM*	YES	free	local	Windows, Linux, MacOS/Matlab	Actinopterygii/other fishes	YES	YES	YES	YES
*Perch*	YES	free	package	TensorFlow	Aves	NA	NA	NA	YES
*PNW-Cnet *	YES	free	local	R + Shiny	Aves	NA	NA	NA	YES
*PorCC*	YES	free	package	Matlab	Certartiodactyla/Pinnipedia	NA	NA	NA	YES
*PortListen*	YES	commercial	online	web browser	Certartiodactyla/Pinnipedia	YES	NA	YES	YES
*Praat*	NO	free	local	Windows, MacOS, Linux, Raspberry, Chromebook	Generalist/multiple taxa	NA	YES	YES	YES
*prinia-project *	YES	free	package	Python, Matlab	Aves	NA	NA	NA	YES
*Pumilio*	YES	free	online	web browser	Generalist/multiple taxa	YES	NA	YES	YES
*pyAudioAnalysis*	NO	free	package	Python	Generalist/multiple taxa	NA	NA	YES	YES
*pykanto*	YES	free	package	Python	Generalist/multiple taxa	NA	YES	YES	YES
*pypam*	YES	free	package	Python	Certartiodactyla/Pinnipedia	NA	YES	YES	YES
*PySoundFile*	NO	free	package	Python	Generalist/multiple taxa	YES	NA	NA	NA
*Python (custom code)*	NO			Python		NA	NA	NA	NA
*PyWavelets*	NO	free	package	Python	Generalist/multiple taxa	NA	YES	NA	YES
*QUT Ecoacoustics Analysis Software*	YES	free	local	Windows, Linux, MacOS	Generalist/multiple taxa	NA	NA	YES	YES
*R (custom code)*	NO			R		NA	NA	NA	NA
*Raven Lite*	YES	free	local	Windows, Linux, MacOS	Generalist/multiple taxa	NA	YES	YES	YES
*Raven Pro*	YES	commercial	local	Windows, Linux, MacOS	Generalist/multiple taxa	NA	YES	YES	YES
*REAL*	YES	free	online	web browser	Generalist/multiple taxa	YES	NA	YES	YES
*Realtime Acoustic Detection daemon (RADd)*	YES	free	local	Linux	Generalist/multiple taxa	NA	NA	NA	YES
*Reaper*	NO	free to try	local	Windows, Linux, MacOS	Generalist/multiple taxa	NA	YES	YES	NA
*RIBBIT*	YES	free	package	R, Python	Amphibia	NA	NA	YES	YES
*ROCCA*	YES	free	package	PamGuard	Certartiodactyla/Pinnipedia	NO	YES	YES	YES
*SaTScan*	NO	free	local	Windows, MacOS, Linux	Chiroptera	NA	NA	NA	YES
*SCAN'R*	YES	commercial	local	Windows	Chiroptera	NA	NA	YES	YES
*scikit-maad *	YES	free	package	Python	Generalist/multiple taxa	YES	YES	YES	YES
*SciPy*	NO	free	package	Python	Generalist/multiple taxa	NA	YES	YES	NA
*SDEer*	YES	free	package	Matlab	Aves	NA	YES	NA	YES
*SeaPro*	YES	free	local	Windows, Linux, MaxOS	Certartiodactyla/Pinnipedia	YES	NA	YES	NA
*Seewave*	YES	free	package	R	Generalist/multiple taxa	YES	YES	YES	YES
*SEISAN*	YES	free	local	Windows, Linux, MacOS	Generalist/multiple taxa	YES	NA	YES	YES
*Sierra Birds*	YES	free	package	TensorFlow	Aves	NA	NA	NA	YES
*SIGNAL*	YES	commercial	local	Windows	Generalist/multiple taxa	NA	NA	YES	YES
*Silbido*	YES	free	package	Matlab	Generalist/multiple taxa	NA	NA	NA	YES
*SILIC*	YES	free	package	Python	Aves	NA	YES	YES	YES
*Sinax*	YES	free	package	R	Generalist/multiple taxa	NA	NA	NA	YES
*Sonic Annotator*	YES	free	local	Windows, Linux, MacOS	Generalist/multiple taxa	NA	NA	NA	YES
*SonicVisualiser*	NO	free	local	Windows, Linux, MacOS	Generalist/multiple taxa	NA	NA	YES	YES
*Sonobat*	YES	commercial	local	Windows, MacOS	Chiroptera	YES	YES	YES	YES
*Sonothèque of the Muséum national d'Histoire naturelle (Paris)*	YES	free	online	web browser	Generalist/multiple taxa	YES	NA	YES	NA
*Sound Analysis Pro*	YES	free	local	Windows	Aves	YES	NA	YES	YES
*SoundClass*	YES	free	package	R	Generalist/multiple taxa	NA	YES	YES	YES
*SoundCoop Portal*	YES	free	online	web browser	Certartiodactyla/Pinnipedia	YES	NA	YES	YES
*Soundecology*	YES	free	package	R	Generalist/multiple taxa	NA	NA	NA	YES
*soundevent*	YES	free	package	Python	Generalist/multiple taxa	YES	YES	YES	YES
*Soundexplorer*	YES	free	local	Windows, Linux, MacOS	Generalist/multiple taxa	NA	NA	NA	YES
*soundfile*	NO	free	package	Python	Generalist/multiple taxa	YES	NA	NA	NA
*SoundFinder*	YES	free	package	R, spreadsheet programs	Generalist/multiple taxa	NA	NA	NA	YES
*Soundgen*	NO	free	package	R	Generalist/multiple taxa	NA	YES	YES	NA
*SoundRuler*	NO	free	local	Linux, Mac, WIndows	Generalist/multiple taxa	NA	NA	YES	YES
*Soundscape Viewer toolbox*	YES	free	package	Matlab	Generalist/multiple taxa	NA	NA	YES	YES
*soundscape_IR*	YES	free	package	Python	Generalist/multiple taxa	NA	NA	YES	YES
*SoundScapeExplorer*	YES	free	local	Windows,Linux, MacOS	Generalist/multiple taxa	NA	NA	YES	YES
*SoundScope*	YES	free	package	Python	Generalist/multiple taxa	YES	NA	YES	YES
*SoundShape*	YES	free	package	R	Generalist/multiple taxa	NA	NA	YES	YES
*SoX*	NO	free	local	Windows, Linux, MacOS	Generalist/multiple taxa	NA	YES	NA	NA
*Sox-o-matic*	YES	free	local	Windows, MacOS	Generalist/multiple taxa	YES	YES	NA	NA
*STUBB*	YES	free	package	PAMGuard, Ishmael	Generalist/multiple taxa	NA	NA	NA	NA
*Switch Plus Audio File Converter*	NO	free	online	web browser	Generalist/multiple taxa	YES	YES	NA	NA
*Syrinx*	NO	free	local	Windows	Generalist/multiple taxa	NA	YES	YES	YES
*Tadarida*	YES	free	local	Windows, Linux	Chiroptera	NA	YES	YES	YES
*Tethys*	YES	free	local	Windows, Linux	Generalist/multiple taxa	YES	NA	NA	YES
*Tierstimmenarchiv*	YES	free	online	web browser	Generalist/multiple taxa	YES	NA	NA	NA
*Time-Frequency Toolbox*	NO	free	package	Matlab	Generalist/multiple taxa	NA	YES	NA	YES
*Triton*	YES	free	local	Matlab, Windows	Certartiodactyla/Pinnipedia	NA	NA	YES	YES
*TuneR*	NO	free	package	R	Generalist/multiple taxa	NA	YES	NA	NA
*TweetyNet*	YES	free	package	Python	Aves	NA	YES	YES	YES
*Vesper*	YES	free	package	Python	Aves	YES	NA	NA	NA
*VGGish*	NO	free	local	TensorFlow	Generalist/multiple taxa	NA	NA	NA	YES
*Vocal Repertoire Embedder*	YES	free	package	Python	Generalist/multiple taxa	NA	NA	NA	YES
*VocalMat*	YES	free	package	Matlab	Other Mammalia	NA	YES	YES	YES
*vocalpy*	YES	free	package	Python	Generalist/multiple taxa	NA	YES	YES	YES
*VoiceLab*	YES	free	package	Python	Other Mammalia	NA	YES	YES	YES
*VoiceSauce*	NO	free	package	Matlab	Other Mammalia	NA	NA	YES	YES
*warbleR*	YES	free	package	R	Generalist/multiple taxa	YES	YES	YES	YES
*WASIS – Wildlife Animal Sound Identification System*	YES	free	local	Windows	Generalist/multiple taxa	YES	YES	YES	YES
*WaveLab*	NO	commercial	local	Windows, MacOS	Generalist/multiple taxa	YES	YES	YES	YES
*Waveman*	YES	free	package	Python	Chiroptera	NA	NA	NA	YES
*WavePad*	NO	free	local	Windows, MacOS	Generalist/multiple taxa	NA	YES	YES	YES
*wavesurfer.js*	NO	free	online	JavaScript	Generalist/multiple taxa	NA	YES	YES	YES
*Whombat*	YES	free	online	web browser	Other Mammalia	YES	YES	YES	YES
*Wildtrax*	YES	free	online	web browser	Generalist/multiple taxa	YES	YES	YES	YES
*XenoCanto*	YES	free	online	web browser	Aves	YES	NA	YES	NA

Software preferences diverged notably between realms ( Figure 3B). We found that terrestrial PAM research dominated the literature, comprising 476 studies. In those studies, *Raven Pro* was most frequently mentioned (95 of 647 total software mentions; 14.7%), followed by custom scripts (e.g., R, Matlab, Python; 72/647; 11.1%) and *Kaleidoscope Pro* (41/647; 6.3%). Tools like *Kaleidoscope* and *BirdNET Analyzer* are particularly relevant to automated species identification and tailored to land-based species, including birds and bats. Other recurrent tools included *Audacity*, *Soundecology*, and *Avisoft SASLab Pro*, which are frequently used for visualization, manual annotation, and basic acoustic analysis.

In contrast, aquatic studies were represented by 319 studies, where custom scripts dominated the software landscape (81/321; 25.2%), followed by *Raven Pro* (37/321; 11.5%) and *PAMGuard* (27/321; 8.4%). *PAMGuard* and other frequently mentioned software such as *Triton*, *Ishmael*, and *C-POD* illustrate a clear functional specialization within the PAM landscape: these tools were primarily developed for underwater applications, focusing on the detection and localization of aquatic, especially marine taxa. Across 64 general or cross-realm studies, software mentions were more heterogeneous and showed no dominant preference. Custom scripts (9/66; 13.6%), *Raven Pro* (4/66; 6.1%), and *Audacity* (3/66; 4.5%) were mentioned most frequently. Some multi-purpose tools, including *Seewave*, *Audacity*, and *Soundecology*, appeared across multiple realms, illustrating their adaptability for general ecoacoustic analyses. Notably, *Arbimon* has recently gained traction, particularly for terrestrial datasets, showing an increasing presence in peer-reviewed studies between 2018 and 2024.

Taken together, these patterns underscore both the breadth and fragmentation of the ecoacoustic software landscape. The proliferation of tools illustrates the vibrant and rapidly evolving nature of ecoacoustics but also reveals a critical challenge: workflow atomisation ( Royaux et al., 2025). Many tools are highly specialized, designed for narrow analytical purposes rather than integrated, and generalized, making them easily reusable into different workflows. While specialization enables tailored solutions to specific research questions, it can also limit interoperability, knowledge transfer, and reproducibility across projects and ecological realms. Such fragmentation has been identified as a recurring obstacle in recent ecoacoustic reviews (e.g., Ross et al., 2023), where the duplication of effort and lack of common standards hinder methodological convergence.

*Raven Pro*’s prominence across realms highlights its cross-domain adaptability and widespread acceptance in different research fields. Even though most tools are open-source or free to use, commercial software remain more frequently employed in published research, largely due to their intuitive graphical user interfaces (GUI), stability, and dedicated user support ( Browning et al., 2017). Such tools, particularly *Raven Pro* and *Kaleidoscope Pro*, enable researchers with limited programming experience to engage in acoustic analyses efficiently. In contrast, many open-source alternatives require higher computational literacy and lack centralized documentation or maintenance, which can discourage use despite their accessibility. This pattern is also evident in the widespread use of self-written custom scripts, especially in marine sciences. It reflects a broader community preference for flexible, researcher-driven workflows when existing tools fail to meet specialized analytical needs. While this flexibility fosters innovation, it also exacerbates reproducibility gaps when those scripts are undocumented, project-specific, and not publicly shared. The coexistence of highly specialized commercial software and bespoke scripting approaches thus mirrors a field that is simultaneously technically advanced yet methodologically disjointed. Addressing this tension by promoting interoperable frameworks, transparent documentation, and open development practices that enable seamless data exchange between different stages of PAM workflows, will be central to advancing ecoacoustic research.

In terms of the deployment form of the software (whether it is running on local machines, on web-servers or integrated within other, usually local software, see Table 1), the majority of tools (n = 100) were developed as package-based frameworks within established programming environments such as R, Python, and MATLAB (e.g., *seewave, soundecology, librosa, Triton*). The prevalence of packages reflects their flexibility, modularity, and adaptability to diverse analytical needs. Such frameworks allow researchers to combine functions, customize workflows, and integrate acoustic analysis with statistical and visualization routines in a reproducible coding environment, thereby addressing multiple functional components of the PAM data workflow. In comparison, 84 tools operated as local desktop applications, providing graphical user interfaces (GUIs) that facilitate data exploration and analysis without the need for programming expertise. These tools, such as *Raven Pro* or *Kaleidoscope Pro* remain widely used because they offer intuitive interfaces, built-in visualization capabilities, and stable offline performance. However, local installations frequently involve manual data handling, with users generally importing, organizing, and processing recordings independently rather than relying on automated or centralized systems. This can constrain scalability for long-term or distributed monitoring efforts, as such tools are less suited for handling large datasets (e.g., multi-terabyte PAM archives) or supporting collaborative, multi-user projects ( Roch et al., 2016; Sugai et al., 2019; Gibb et al., 2019).

A smaller number of tools (n = 37) were available as web-based platforms like *Arbimon*, or *BirdNET Analyzer.* Although other platforms like *APLOSE* ( Dubus et al., 2025) for aquatic acoustic data exist, they were underrepresented - likely because many marine workflows still rely on locally installed, custom pipelines, reflecting large file sizes and limited bandwidth at sea. These online systems support remote access, collaborative annotation, and centralized data storage, representing an important step toward integrative ecoacoustic infrastructures. Yet, their long-term sustainability depends on reliable server maintenance, stable internet connections, and predictable funding: a particular challenge for tools developed within academic projects, where financial and technical support often ends once project funding ends. However, the diversity of deployment modes illustrates a continuum between flexibility and usability: packages maximize analytical control and reproducibility, whereas GUI-based and web-based systems prioritize accessibility and user-friendliness. Moving forward, the development of open, modular, and platform-independent architectures, capable of operating both locally and in the cloud, will be essential to meet the growing analytical and collaborative demands of the ecoacoustic community.

### 3.2 PAM workflow components and their coverage by existing software tools

To better understand user needs and development priorities, we examined how existing software tools are distributed across the four workflow components used in PAM: Data Management (DM), Signal Pre-processing (SP), Visualisation and Navigation (VN), and Acoustic Analysis (AA) ( Figure 3C). These classes capture the principal components of the ecoacoustic workflow, from raw data handling to interpretation of biological and ecological patterns. Our results show that functional coverage across the 221 available software tools was highly uneven, with 39 tools offering all four functionality components ( Figure 3C). Among the most comprehensive and frequently mentioned in PAM studies were *Arbimon*, *Kaleidoscope Pro*, and *PAMGuard.* Each provides the full suite of the PAM workflow categories from data management to acoustic analysis and thereby providing end-to-end analytical capacity. Most other tools, however, focused on one or two workflow components, underscoring fragmented and specialized solutions within the current software landscape available for ecoacoustic research. In the following subsections (3.2.1–3.2.4) we assess the extent to which each workflow component is covered by existing tools, along with current limitations and future development needs.

3.2.1 Data management

Software tools with functionalities designed for data management were the least represented across all tools ( Figure 3C). Only 92 software tools out of 221 were offering dedicated data handling capabilities. Data management commonly co-occurred together with visualisation and navigation and acoustic analysis (25 tools), excluding signal pre-processing. This is striking given that passive acoustic monitoring produces vast numbers of audio files (often amounting to several terabytes per project), and while technically simple, require extensive organization, metadata management, and processing infrastructure to remain usable ( Sugai et al., 2019; Pijanowski et al., 2024).

Data management in ecoacoustics typically includes structured file organisation, metadata standardisation, automated backup, storage, and the maintenance of public collections: essential processes for ensuring long-term usability and reproducibility ( Roch et al., 2016; Ross et al., 2023). Several factors may explain this limited representation. First, many research institutes and projects have developed their own data management workflows, often tailored to specific infrastructures or institutional requirements, which may have evolved independently of general-purpose PAM software. Second, data management is frequently undervalued compared to analytical components of a workflow, sometimes regarded as a “necessary administrative task” rather than an integral part of scientific analysis. Nonetheless, this perception is beginning to shift as data volumes grow, and reproducibility becomes a central concern. The current lack of integrated solutions forces researchers to rely on ad hoc file structures or external repositories, increasing the risk of data loss and inconsistency. In other ecological monitoring disciplines, adherence to FAIR (Findable, Accessible, Interoperable, Reusable) data principles has already improved transparency and collaboration ( Bayer et al., 2023; Inau et al., 2023; Wilkinson et al., 2016) - similar advances are urgently needed in bio- and ecoacoustics. Achieving such FAIRness begins with consistent metadata standardisation, which forms the foundation for data discoverability and interoperability across systems. Future tool development should therefore prioritise modular data management components: independent yet compatible elements that can be flexibly combined within different workflows, supported by interoperable database interfaces capable of linking seamlessly with analytical and visualisation software (e.g., through standardised APIs or FAIR-compliant data formats). Pioneering initiatives such as Tethys ( Roch et al., 2022) and recent efforts presented at Living Data 2025 (a conference and broader initiative focused on data management, interoperability, and open science in biodiversity and environmental research) exemplify progress toward shared metadata frameworks and harmonised data infrastructures in marine PAM. In contrast, most terrestrial infrastructures remain more taxon-focused, particularly around birds, with community-driven resources such as *xeno-canto* and *BirdNET* leading development ( Vellinga and Planqué, 2015; Wood et al., 2022), but dedicated, cross-project metadata systems for ecoacoustics remain rare.

3.2.2 Signal pre-processing

Signal pre-processing functionality was offered in 100 software tools and commonly co-occurred with visualisation and navigation and acoustic analysis functions (35 tools; Figure 3C). Pre-processing functions typically include amplification, extraction and segmentation, filtering, denoising, and temporal or spectral normalization: essential operations for improving signal quality before analysis. Despite its importance, pre-processing was often implemented as an auxiliary module rather than an integrated workflow component, with limited options for automation or batch handling.

The strong representation of pre-processing functionalities reflects the technical maturity of ecoacoustic workflows at the signal-cleaning component but also highlights methodological inconsistencies: different tools apply distinct filtering algorithms, frequency thresholds, and normalization schemes, often without standardized documentation. Notably, signal preprocessing tends to be more intensive in aquatic studies, where propagation effects (e.g. frequency-dependent attenuation, reverberation), high ambient noise, and corrections for hydrophone sensitivity and deployment depth require more sophisticated filtering, calibration, and noise-reduction steps (e.g. Bittle and Duncan, 2013; Merchant et al., 2015). In contrast, terrestrial frameworks primarily emphasise calibration for estimating detection ranges (e.g. Darras et al., 2016). Such variability complicates cross-study comparability and the creation of shared reference datasets. Future development efforts should therefore emphasize transparent algorithm reporting, parameter standardization through consistent units, and reporting formats, and interoperability between pre-processing and downstream analytical modules to ensure reproducible data pipelines.

3.2.3 Visualisation and navigation

Visualisation and navigation functions were present in a substantial subset of 139 tools, most frequently coupled with core functionalities excluding data management (35 tools, Figure 3C). These tools typically provide spectrogram and waveform displays, temporal and frequency navigation, as well as playback interfaces.

The prevalence of visualisation and navigation capabilities reflects the central role of spectrogram-based inspection in ecoacoustic workflows, both for human interpretation and because most deep-learning classifiers (models) for sound classification operate on spectrogram representations. Despite advances in automated pipelines, manual spectrogram review remains essential for quality control, for creating and verifying the labelled examples used to train and validate models, and for interpreting complex or context-dependent acoustic events (e.g., Gibb et al., 2019; Kitzes et al., 2025; Priyadarshani et al., 2018). However, most visualization tools remain desktop-based and optimized for relatively small datasets, limiting their scalability for long-term or networked monitoring efforts. Cloud-based visualisation environments and integrated dashboards could help overcome these constraints, enabling collaborative annotation and real-time data exploration ( Darras et al., 2024).

3.2.4 Acoustic analysis

Acoustic analysis, covered by 191 tools, was by far the most common functionality component among all tools and dominated the PAM software landscape. While 47 tools were dedicated exclusively to acoustic analysis, many others combined it with one or more additional workflow components ( Figure 3C). Many analytical tools incorporate automated detection, pattern recognition, or machine learning algorithms, such as *BirdNET Analyzer*, *Arbimon*, *PAMGuard*, and *Kaleidoscope Pro* - providing pre-trained models for specific taxa.

This dominance illustrates the field’s strong emphasis on species identification, and sound classification or acoustic index-based interpretation ( Bradfer-Lawrence et al., 2023), reflecting the central role of analysis in translating acoustic signals into ecological information. While this focus demonstrates technical sophistication and innovation, it also reveals a developmental bias: analytical capacities have advanced faster than supporting processes like data management or visualization. The rapid adoption of AI-based (deep-learning) classifiers is transforming ecoacoustic research, enabling large-scale analyses with increasing accuracy (e.g., Kahl et al., 2021; Lauha et al., 2022; Priyadarshani et al., 2018). However, these approaches rely heavily on curated training datasets and transparent model validation, both still limited in scope and accessibility. Future progress will depend on open benchmark datasets, cross-taxa model sharing, and standards for performance reporting to ensure comparability and ecological interpretability of analytical results.

## 4. Conclusion

Our analysis highlights both the remarkable diversity and the pronounced fragmentation of the ecoacoustic software landscape. With 251 identified tools, of which 174 were explicitly designed for passive acoustic monitoring, the field shows clear signs of technical maturity but also considerable redundancy. The proliferation of software tools, many of which likely replicate similar workflow components, reflects both the creativity and decentralization that characterize this field. However, this redundancy, combined with the fact that most tools do not cover all functionality components of the PAM data workflow, underscores the inefficiency and lack of interoperability that currently limit analytical coherence. At the same time, the growing number of PAM-dedicated tools marks a positive trend toward specialized solutions capable of addressing the technical demands of passive acoustic monitoring. Emerging tools are increasingly integrating machine learning algorithms for automated detection, classification, and even ecological inference, enabling large-scale soundscape analyses that were previously unfeasible. To build on this progress, future developments should prioritise connecting or integrating existing tools, rather than multiplying them through isolated efforts. Future PAM data infrastructures will need to incorporate FAIR-aligned data management, standardized metadata frameworks, and open benchmark datasets to ensure that core functionalities remain transparent, reproducible, and ecologically meaningful. In practice, this requires active collaboration between software developers, ecologists, and data scientists to connect the current ecosystem of specialized tools into flexible, modular platforms capable of scaling across realms, taxa, and temporal or spatial resolutions.

## Declaration of generative AI and AI-assisted technologies in the writing process

During the preparation of this work, the authors used ChatGPT (OpenAI) in order to assist with language editing and improve the clarity of the text. After using this tool, the authors reviewed and edited the content as needed and take full responsibility for the content of the publication.

## Data Availability

The dataset, PRISMA checklist and R script underlying this study are available on the Open Science Framework (OSF): https://doi.org/10.17605/OSF.IO/V72CE, ( Hanf-Dressler et al., 2025). This project contains the following underlying data:
• PRISMA_CHECKLIST&FLOWCHART.pdf - Complete PRISMA checklist (2020) and flowchart diagram ( Figure 1). •
ECOACOUSTIC_SOFTWARE_INVENTORY.xlsx – Comprehensive list of 251 software tools used in ecoacoustic and bioacoustic studies, including software metadata and main functionality components in PAM workflows. •
ECOACOUSTIC_SOFTWARE_DENDROGRAM.html – Interactive dendrogram visualizing the software landscape by metadata and functionality characteristics. •
PAM_SOFTWARE_ANALYSIS.R – Main analysis script, including data import, processing, and generation of figures included in the manuscript. Data are available under the terms of the Creative Commons Zero “No rights reserved” data waiver (CC0 1.0 Public domain dedication).
