# Supplementary material for: Software Tools for Passive Acoustic Monitoring in Aquatic and Terrestrial Bio- and Ecoacoustics: A Living Systematic Review
Source: F1000Res. 2026 Jan 13;15:48. [Version 1] doi: 10.12688/f1000research.173495.1 (PMC13096786; doi:10.12688/f1000research.173495.1)
Supplement: Peer review history

# Reviewer report: Reviewer response for version 1

**DOI:** 10.5256/f1000research.191317.r453949

**Published:** 2026-04-20

**Authors:** Tessa Rhinehart

**Affiliations:** 1 Department of Biological Sciences, University of Pittsburgh, Pittsburgh, Pennsylvania, USA

I was glad to get a chance to review this paper! It represents a significant expansion of current databases. I thought the text was well-written and hit upon important points, especially the importance of standardized frameworks, metadata, etc.

 I ask the authors to address just a few comments to improve the usability of this Living Review. First, the software table could use some additional details - at least URLs/DOIs for each piece of software that is still available online. Much of the software listed is hard to find online based on its name alone. 

 Second, since one of the primary goals of the review is to help bio acousticians select tools, the authors should describe the specific functionality of each software. The categories used are, in my opinion, too broad to be helpful for software selection – for example, “Acoustic Analysis” includes template matching, annotation, acoustic index calculation, and machine learning classifier training. Also, I suggest that “annotation” belongs in its own category separate from analyses.

 Third, since the review will be updated biannually, can the authors please describe the planned update procedure? Rerunning the search, re-reviewing the software webpages, and updating links and descriptions of all software in the spreadsheet? If applicable, what is the funding mechanism through which updates will be supported or guaranteed? 

 Cheers,

 Tessa Rhinehart

1. Is the work clearly and accurately presented and does it cite the current literature?

Yes

Are the rationale for, and objectives of, the Systematic Review clearly stated?

Yes

Is the statistical analysis and its interpretation appropriate?

Yes

If this is a Living Systematic Review, is the ‘living’ method appropriate and is the search schedule clearly defined and justified? (‘Living Systematic Review’ or a variation of this term should be included in the title.)

Partly

Are sufficient details of the methods and analysis provided to allow replication by others?

Partly

Are the conclusions drawn adequately supported by the results presented in the review?

Yes

Reviewer Expertise:

bioacoustics methodological development

I confirm that I have read this submission and believe that I have an appropriate level of expertise to confirm that it is of an acceptable scientific standard, however I have significant reservations, as outlined above.

# Reviewer report: Reviewer response for version 1

**DOI:** 10.5256/f1000research.191317.r453943

**Published:** 2026-02-28

**Authors:** Santiago Martinez Balvanera

**Affiliations:** 1 University College London, London, UK

This work clearly identifies an important gap in understanding how bioacoustic and ecoacoustic research is conducted, particularly regarding the software tools that support these workflows.

 The review is thorough and provides a valuable resource for the community.

 The results offer a broad overview of the rapidly expanding ecoacoustic tool landscape and highlight opportunities for greater workflow standardisation.

 1. The proposed classification is a useful starting point, but two aspects could be strengthened.

    First, the categories are implicitly defined through examples and the provided discussion; making these definitions explicit at the outset would help clarity.

    Second, additional justification for the classification scheme would be helpful.

    Some categories, particularly “Acoustic Analysis", are quite broad, which may limit their usefulness for readers seeking tools that match specific needs.

    A more granular breakdown could help navigation within the 200+ tools presented.

 2. Some minor methodological details are missing: How were query specificity and relevance assessed during iterative refinement of the search string?

    Was screening conducted independently or blindly by multiple authors?

    How were disagreements resolved?

    Clarifying these points would help transparency and reproducibility.

 3. The software list provided is highly useful.

    Consider adding a column with links to each tool's homepage where available.

 4. Figure 3 is well designed and polished, but the colour legend in panel B appears to be missing.

    I found panel C difficult to interpret, particularly the connected dots section, and the caption does not fully explain it.

    A brief clarification in the caption would improve readability.

Are the rationale for, and objectives of, the Systematic Review clearly stated?

Yes

Is the statistical analysis and its interpretation appropriate?

Yes

If this is a Living Systematic Review, is the ‘living’ method appropriate and is the search schedule clearly defined and justified? (‘Living Systematic Review’ or a variation of this term should be included in the title.)

Yes

Are sufficient details of the methods and analysis provided to allow replication by others?

Partly

Are the conclusions drawn adequately supported by the results presented in the review?

Yes

Reviewer Expertise:

bioacoustics, software development, machine learning

I confirm that I have read this submission and believe that I have an appropriate level of expertise to confirm that it is of an acceptable scientific standard.

# Reviewer report: Reviewer response for version 1

**DOI:** 10.5256/f1000research.191317.r453942

**Published:** 2026-02-26

**Authors:** Daniel Alexis Nieto Mora

**Affiliations:** 1 Instituto Tecnológico Metropolitano ITM, Medellín, Colombia

The submitted work presents an important systematic review of a wide variety of tools used for Passive Acoustic Monitoring (PAM). It is evident that substantial background work has been conducted, with methodological rigor following PRISMA and presenting, at a good level of detail, the processes of acquisition, review, pre-selection (screening), and selection of the final articles included in the analysis. In this sense, the work appears well-focused and demonstrates a clear vision of its objectives and intended contributions.

 As a positive aspect, I particularly highlight the consideration of the four main PAM approaches: data management, signal pre-processing, spectrogram visualization and navigation, and acoustic analysis. This structure provides a clear and logical framework that facilitates understanding of the different functional dimensions of PAM tools and contributes to the overall coherence of the review.

- In the first paragraph of the Methods section, it is stated that the software tools considered span from 2014 to January 2024. This temporal scope excludes the most recent tools developed over the past two years. Given that this is a review study, where priority is generally placed on the inclusion of the most up-to-date information, this decision should be thoroughly justified.
- In Section 2.1 (Literature Review), further clarification is needed regarding how the final review of the selected studies was conducted. Specifically, it would be important to indicate how many reviewers participated, whether any validation process was applied, whether specialized review software was used, and to clearly define the exclusion criteria.
- The four main components analyzed are mentioned repeatedly throughout the text. It would be advisable to assess whether such emphasis is necessary. If not, the references to these components could be consolidated and presented in the most appropriate section to avoid redundancy.
- Although there is an exhaustive analysis of the taxa reported in the studies, there is limited discussion or graphical representation of this information. While I understand that the primary focus of the article is not on species per se, the review process has already generated valuable data in this regard. Therefore, it would be beneficial to further develop and make better use of this information.
- Overall, I consider that the manuscript contains relatively few figures to support the work. Including a few additional graphs to strengthen the Results and Discussion sections would enhance the manuscript. Given the exhaustive and rigorous nature of the review, there is certainly sufficient material available to visually highlight some of the most relevant findings.
- Table 2 is of central importance to the manuscript. However, it may be worth considering whether it could be reformatted vertically to improve readability, possibly by presenting the pages in landscape orientation to allow for wider margins and better visualization.

Are the rationale for, and objectives of, the Systematic Review clearly stated?

Partly

Is the statistical analysis and its interpretation appropriate?

Partly

If this is a Living Systematic Review, is the ‘living’ method appropriate and is the search schedule clearly defined and justified? (‘Living Systematic Review’ or a variation of this term should be included in the title.)

Partly

Are sufficient details of the methods and analysis provided to allow replication by others?

Yes

Are the conclusions drawn adequately supported by the results presented in the review?

Yes

Reviewer Expertise:

Machine learning applied to ecoacoustics and bioacoustics or passive acoustic monitoring.

I confirm that I have read this submission and believe that I have an appropriate level of expertise to confirm that it is of an acceptable scientific standard, however I have significant reservations, as outlined above.
